# Hsa_circ_0074158 regulates the endothelial barrier function in sepsis and its potential value as a biomarker

**DOI:** 10.3389/fgene.2022.1002344

**Published:** 2022-11-08

**Authors:** Haiyan Liao, Yan Chai, Yuming Sun, Zhe Guo, Xuesong Wang, Ziyi Wang, Ziwen Wang, Zhong Wang

**Affiliations:** ^1^ School of Clinical Medicine, Tsinghua University, Beijing, China; ^2^ Clinical Laboratory, Beijing Tsinghua Changgung Hospital, School of Clinical Medicine, Tsinghua University, Beijing, China; ^3^ General Department, Beijing Tsinghua Changgung Hospital, School of Clinical Medicine, Tsinghua University, Beijing, China

**Keywords:** barrier function, RNA-seq, CircRNAs, adherens junction, sepsis

## Abstract

**Background:** Sepsis is one of the main causes of death in critically ill patients with high morbidity and mortality. Circular RNAs (CircRNAs) are aberrantly expressed, and play significant regulatory roles in many diseases. However, the expression profiles and functions of circRNAs in sepsis have not yet been fully clarified.

**Methods:** Our present study performed an RNA sequencing (RNA-seq) analysis to assess the expression profiles of circRNAs *in vitro*. We applied the quantitative real-time polymerase chain reaction (RT-qPCR) to verify the RNA-seq results. The analyses of Gene Ontology (GO), Kyoto Encyclopedia of Genes and Genomes (KEGG) pathway, the competitive endogenous RNA (ceRNA) regulatory networks, were performed to explore the potential mechanism in sepsis. And then, significantly up-regulated differentially expressed (DE) circRNA, hsa_circ_0074158, was selected for further study. Hsa_circ_0074158 was silenced to investigate its regulatory function in sepsis, and the barrier function was also examined *in vitro*. Endothelial cell junctions were valued using Vascular endothelial cadherin (VE-cadherin), which was detected by immunofluorescence staining. We measured endothelial permeability by transendothelial electrical resistance (TEER) and fluorescein isothiocyanate (FITC)-dextran extravasation.

**Results:** In total, 203 significantly DE circRNAs, including 77 up-regulated and 126 down-regulated, were identified. *In vitro,* the RT-qPCR assay showed that the expression pattern of hsa_circ_0074158, hsa_circ_RSBN1L_11059, hsa_circ_0004188, and hsa_circ_0005564 were consistent with the results from RNA-seq analysis. The expression of hsa_circ_0074158 detected by RT-qPCR *in vivo* was also consistent with the RNA-seq results. The ceRNA networks, GO enrichment, and the KEGG pathway analyses revealed that circRNAs may be related to the barrier function in sepsis. The immunofluorescence assay showed that the suppression of hsa_circ_0074158 expression significantly enhanced the expression of VE-cadherin, which was suppressed in lipopolysaccharide (LPS)-induced sepsis. Additionally, hsa_circ_0074158 knockdown could partially reverse the LPS-induced TEER reduction and FITC-dextran extravasation elevation in sepsis.

**Conclusion:** In conclusion, we have found DE circRNAs could serve as potential biomarkers and therapeutic targets for sepsis. Hsa_circ_0074158 plays a vital role in sepsis and is related to the disruption of the endothelial barrier.

## 1 Introduction

### 1.1 Background

Sepsis is defined as “life-threatening organ dysfunction caused by a dysregulated host response to infection” (Singer et al., 2016). Sepsis is also considered one of the leading causes of death in critically ill patients with high morbidity and mortality (Vincent et al., 2014; Machado et al., 2017). Multiple factors are involved in the complex pathophysiological process of sepsis, including impaired vascular endothelial barrier, enhanced vascular permeability, inflammatory response, immune response, and coagulation dysfunction. Sepsis is a serious threat to human health and the prevalence of sepsis is considered a significant burden for health systems. Research from 1979 to 2015 in seven high-income countries showed that morbidity was 288 hospital-treated sepsis cases and 148 hospital-treated severe sepsis cases per 100,000 person-years, while morbidity was 437 sepsis and 270 severe sepsis cases per 100,000 person-years in the last decade, with hospital mortality 17% for sepsis and 26% for severe sepsis (Fleischmann et al., 2016).

The vascular endothelium is composed of a monolayer of endothelial cells, a basement membrane, an extracellular matrix, and endothelial glycocalyx, which lines the luminal surface of the inner blood of vessels with extensive homeostasis functions (Rajendran et al., 2013). Vascular endothelial cadherin (VE-cadherin) is concentrated in adherent junctions and found exclusively in the vascular endothelium in vertebrates, additionally, it also plays an important role in stabilizing the barrier function of the endothelium (Brasch et al., 2011). As a dynamic and heterogeneous organ, the endothelium is involved in many biological functions, such as the secretory, metabolic, and immunologic functions (Rajendran et al., 2013; Godo and Shimokawa, 2017). Through the synthesizing and release of various relaxing factors, such as vasodilator prostaglandins, nitric oxide, hyperpolarization factors, and contracting factors, the endothelium is tightly involved in the regulation of vascular tone (Nava and Llorens, 2019; Shimokawa and Godo, 2020; Godo et al., 2021). Many pathological processes, including microvascular tone dysfunction, vascular permeability, inflammatory response, platelet adhesion and aggregation, coagulation and fibrinolysis, and immunological response, are significantly associated with endothelial dysfunction. The endothelium is considered a critical physical barrier in microcirculation, and in the physiopathological process of sepsis, endothelial dysfunction is a central event (Lee and Slutsky, 2010; Martin-Fernandez et al., 2021).

Circular RNAs (CircRNAs) were discovered for more than 40 years and also observed in the cytoplasmic fractions (Sanger et al., 1976; Hsu and Coca-Prados, 1979). CircRNAs are endogenous noncoding RNA in eukaryotic cell lines that are mainly derived from the pre-mRNAs by back-splicing of exons and are initially considered an aberrant splicing event during the gene transcription (Nigro et al., 1991; Cocquerelle et al., 1992; Cocquerelle et al., 1993; Pasman et al., 1996). Due to lacking polyadenylation [poly(A)] and capping, the 5′ and 3′ splice sites of circRNAs are covalently closed, and this structure determines its highly conservative, specific, and stable properties (Chen, 2016; Kristensen et al., 2019; Chen, 2020). CircRNAs are aberrantly expressed in many diseases and also act as miRNA and protein sponges to be involved in the regulation of different processes in sepsis (Ashwal-Fluss et al., 2014; Thomson and Dinger, 2016; Abdelmohsen et al., 2017; Panda, 2018; Kristensen et al., 2019; Beltran-Garcia et al., 2020; Chen, 2020). An increasing number of studies revealed that circRNAs have been significantly associated with the regulation of many physiological and pathological processes, such as innate immunity, cell proliferation and transformation, and neuronal function (Chen, 2020; Chen et al., 2021). Dysregulation of circRNAs was also observed in various human diseases, including neurogenesis, myogenesis, osteogenesis, cancer, cardiovascular disease, metabolic diseases, and other metabolism-associated diseases (Huang et al., 2021; Verduci et al., 2021). Recent studies also demonstrated the potential values of circRNAs as diagnostic biomarkers and/or therapeutic targets in many diseases (Jiang et al., 2022; Wang et al., 2022; Zhang et al., 2022). Additionally, circRNAs have been reported to be used as new diagnostic biomarkers and molecular therapeutic targets (Tian et al., 2021). However, whether circRNAs are involved in the regulation of the endothelial barrier in sepsis is not fully explored.

Many RNA sequencing (RNA-seq) studies have found that differentially expressed (DE) circRNAs were involved in cellular functions (Chen, 2020; Chen et al., 2021; Huang et al., 2021; Verduci et al., 2021). In this study, we detected the expression profiles of circRNAs in lipopolysaccharide (LPS)-induced sepsis. Then we explored their value as biomarkers in sepsis and further explored the effect of hsa_circ_0074158 on the endothelial barrier.

## 2 Materials and methods

### 2.1 Samples and patients

In order to investigate DE circRNAs in LPS-induced sepsis *in vitro,* the human umbilical vein endothelial cells (HUVECs) were divided into two groups: 1) control group, treated with nothing; 2) LPS group, treated with 1 μg/ml LPS for 12 h. Each sample was collected for three independent biological replicates. The HUVECs came from the Institute of Immunology, Tsinghua University, and were grown in Dulbecco’s modified Eagle’s Medium (DMEM; Solarbio, China) supplemented with 10% fetal bovine serum (FBS; TianHang, China) at 37°C in a humidified atmosphere of 5% CO_2_. LPS was purchased from Sigma-Aldrich (Cat No. L4516). After treatment, cells were washed once with Phosphate buffer saline (PBS; Solarbio, China). Subsequently, the samples were centrifuged at 1,500 rpm for 5 min and stored in a freezer at −80°C.

According to Sepsis −3, 44 patients with sepsis and 48 healthy individuals were enrolled at Beijing Tsinghua Changgung Hospital (Beijing, China) from April 2022 to June 2022. Whole blood samples were collected and the quantitative real-time polymerase chain reaction (RT-qPCR) was performed. The total RNA was immediately extracted using Hipure Blood RNA Kits (Magen, Guangzhou, China). All patients had no history of autoimmune disorders, neoplastic diseases, or oral immunosuppressants. This study was approved by the Ethics Committee (NCT05095324).

### 2.2 Construction of transcriptome libraries

The total RNA of HUVECs was extracted using the total RNA kit (TaKaRa, Japan), followed by the manufacturer’s instructions. The concentration and purity of the extracted RNA were determined using Nanodrop One (Thermo Fisher, United States). The integrity and contamination of RNA were evaluated by agarose gel electrophoresis (Bio-RAD, United States). And the integrity of the RNA was further verified using an Agilent 2100 bioanalyzer (Agilent 2100, United States). The preparation of specific transcriptome libraries was performed by removing ribosomal RNA. RNA was degraded using Ribonuclease (RNase) H (TaKaRa, Japan). The first single-stranded cDNA was synthesized with reverse transcription of RNA, followed by the second double-stranded cDNA synthesized using dNTP (dUTP, dATP, dGTP, and dCTP). Subsequently, RT-qPCR was performed to amplify the sequences and then the preparation of the total RNA library was completed. The RT-qPCR and the Agilent 2100 bioanalyzer were used to control the quality and quantity of the library. Transcriptome sequencing was performed with Illumina PE150.

### 2.3 Sequence alignments

The alignment of valid sequencing data (clean reads) with the genome or transcriptome is the basis for subsequent analysis. We used Hisat2 software (version 2.0.1-beta) to align the transcriptome RNA-seq dataset. Hisat2 software is an upgrade of Tophat2 software, with a high mapping rate and high accuracy in finding Junction reads (Kim et al., 2015).

### 2.4 Differential expression analysis

The input data for the differential expression of circRNAs was the read counts data set obtained from the analysis of circRNA expression. For samples with biological duplications, the DESeq2 package was used for the analysis of miRNAs and circRNAs between the LPS and control groups (Love et al., 2014). For samples without biological duplications, DEGseq provides a TMM algorithm to standardize the read count data and then performs a difference analysis.

### 2.5 Enrichment analysis of differentially expressed circRNAs

After obtaining the DE circRNAs between the LPS and control groups, the host genes in each group were analyzed by enrichment of the Gene Ontology (GO) (version 1.1) and Kyoto Encyclopedia of Genes and Genomes (KEGG) (version 94.0) pathway, respectively. The GO is a major bioinformatics initiative to unify the representation of gene and gene product attributes across all species (Consortium, 2019). KEGG is a collection of databases dealing with genomes, biological pathways, diseases, drugs, and chemical substances (Kanehisa and Goto, 2000). The KEGG PATHWAY database is the core of the KEGG resource. The database is a collection of pathway maps that integrate many entities, including genes, proteins, RNAs, chemical compounds, glycans, and chemical reactions, as well as disease genes and drug targets, which are stored as individual entries in the other KEGG databases. GO enrichment analysis consists of biological process (BP), cellular component (CC), and molecular function (MF), performed to construct gene annotations. The enrichment of the KEGG pathway was carried out to reveal the function and interactions among the DE genes, providing annotation information on signal transduction and disease pathways.

### 2.6 Construction of a regulatory network for the competitive endogenous RNA

CircRNA is known as a miRNA sponge. Through binding to target miRNA, circRNA can inhibit miRNA expression and subsequently affect the ceRNA network. Thus, analysis and identification of the binding sites of circRNAs and their target miRNAs are necessary. In this study, Starbase software (version 2.0) and miRanda software (version 3.3a) were used to predict target miRNAs and potential binding sites with DE circRNAs, and the target genes of predicted miRNAs were identified using miRWalk (version 2.0) (Li et al., 2014; Agarwal et al., 2015; Sticht et al., 2018).

### 2.7 Real-time polymerase chain reaction

The validation of the results obtained from the RNA-seq and the quantification of candidate circRNA expression were performed using RT-qPCR. Specific convergent primers that span the circRNA back-splice junction site were designed by the circPrimer (version 2.0) and Oligo 7 (version 7.37) software (Zhong et al., 2018). If there were no suitable convergent primers, divergent primers were designed similarly. The cDNA was synthesized using the PrimeScript™ RT reagent kit (TaKaRa, Beijing, China). The RT-qPCR was performed using TB Green Premix Ex Taq™ II (TaKaRa, Beijing, China). GAPDH was employed as an internal control. RT-qPCR was performed by three independent biological and technical duplications. The relative expression of the circRNAs was analyzed by the 2^−ΔΔCT^ method.

### 2.8 Immunofluorescence staining

The HUVECs were seeded in 24-well plates and cultured for 24 h. After the indicated treatment, cells were washed with cold PBS and fixed in 4% paraformaldehyde. The cells were then blocked with 5% BSA in PBS for 1 h and incubated with specific primary antibodies (1:100; Abcam) at 4°C overnight. The cells were then incubated with a fluorochrome-labeled anti-rabbit secondary antibody (1:500; Beyotime) for 1 h at room temperature with protection from light. The cells were then washed with cold PBS three times and stained with DAPI (ready-to-use, Solarbio) for nucleus staining. The expression of VE-cadherin was observed by fluorescence microscopy (Olympus).

### 2.9 Transendothelial electrical resistance and fluorescein isothiocyanate -dextran assays

Cultivation of cell monolayers on the upper side of the Transwell insert (Corning, United States) at 37°C, 5% CO_2_. In the upper and lower chambers, 200 μL and 600 µL culture medium were distributed, respectively. After treatment, the Volt-Ohm Meter (Millipore, United States) was used to measure TEER following the protocol. The values (Ω cm^2^) of TEER were expressed by subtracting the resistance of the blank insert and correcting for the surface area.

Culture HUVECs as previously described. After treatment, FITC-dextran (1 mg/ml, Sigma-Aldrich, United States) was added to the upper chambers and cultured for 45 min 100 µL samples were collected from the upper and lower chambers for fluorescence. The dextran permeability coefficient (Pd) was used to evaluate the permeability of the endothelial monolayer.

$$Pd=\frac{\left[A\right]}{t}\times \frac{1}{A}\times \frac{V}{\left[L\right]}$$

[*A*] is the dextran concentration of the lower chamber, *t* is time, *A* is the membrane area; *V* is the volume of the lower chamber [*L*] is the dextran concentration of the upper chamber (Wu et al., 2020).

### 2.10 Statistical analysis

SPSS software (version 26.0) was used to analyze all the data collected from this study and the figures were produced using GraphPad Prism (version 8.0.2). Comparisons between two groups were analyzed using the student’s t-test and comparisons between multiple groups were performed by one-way ANOVA. All data are presented as mean ± standard deviation (S.D.). The chi-square test or Fisher’s exact test was applied to analyze the categorical variables. The *p*-value <0.05 was considered statistically significant.

## 3 Results

### 3.1 RNA-seq identified features of circRNAs and differentially expressed profiles

**FIGURE 1:**
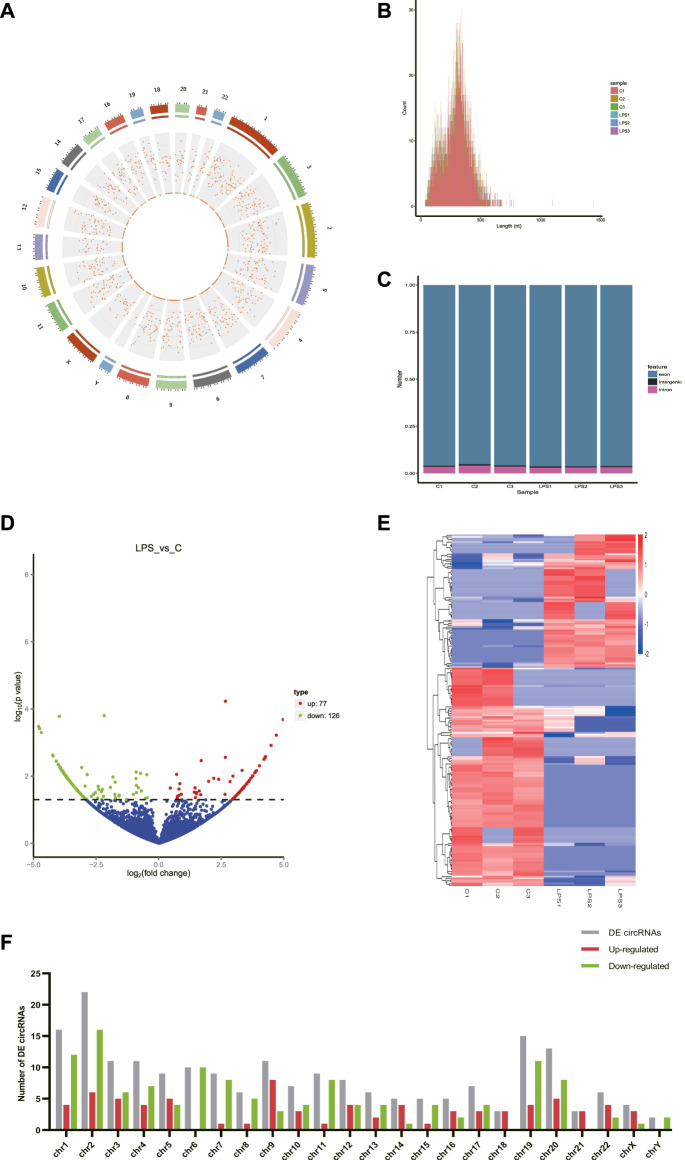
CircRNAs in LPS-induced sepsis. **(A)** The distribution of all cricRNAs in sepsis. The outermost layer was a chromosome map of the human genome. The red dots in the inner circles represented the circRNAs. **(B)** The length of the circRNA of all samples and the source of the circRNAs were represented. **(C)** The source of all circRNAs. **(D)** Representative volcano plot showing the up- and down-regulated circRNAs in the cells treated with or without LPS. Red dots represent up-regulated transcripts, green dots represent down-regulated transcripts, and blue dots represent genes without a significant change. **(E)** Cluster analysis of all differentially expressed transcripts. Up- and down-regulated circRNAs are colored red and blue, respectively. **(F)** Distribution of DE circRNAs on chromosomes. **(C)** Control groups. LPS: LPS groups.

A total of 8766 circRNAs were identified by RNA-seq. The distribution of all cricRNAs on human chromosomes was analyzed and the results were shown in Figure 1A. Meanwhile, the length of the circRNAs of all samples and the source of these circRNAs were shown in Figures 1B,C, respectively. According to Figure 1B, we found that the length of most circRNAs in HUVECs is less than 500 nt. Figure 1C revealed that circRNAs can be derived from exons, introns, and intergenic, but most of the circRNAs in HUVECs are derived from the exon.

According to the filtering conditions of |log_2_ (FoldChange)| > 1 and the *p*-value <0.05, 203 circRNAs were identified as significantly DE circRNAs (77 circRNAs were up-regulated and 126 were down-regulated). Based on the top dysregulated circRNAs, 20 circRNAs were selected from those identified DE circRNAs (Table 1). To verify the results of the RNA-Seq analysis, the expression of the selected 20 DE circRNAs was verified using RT-qPCR. The significant differences in DE circRNAs between LPS and control groups were shown with a volcano plot (Figure 1D) and cluster analysis (Figure 1E). As shown in Figure 1F, sepsis-associated circRNAs were distributed on each chromosome, while more DE circRNAs were located on chromosome 2.

**TABLE 1:** The 20 DE circRNAs of the RNA-seq dataset.

CircRNA ID	Chromosome	Feature	Gene symbol	Regulation	log2FoldChange	*p*-value
hsa_circ_0002360	21	exon	RUNX1	Up	5.0417	0.0001
hsa_circ_0069338	4	exon	SEPSECS	Up	4.9572	0.0002
hsa_circ_0007816	9	exon	UHRF2	Up	4.6863	0.0006
hsa_circ_0074158	5	exon	CTNNA1	Up	4.4764	0.0012
hsa_circ_TRAK1_8343	3	exon	TRAK1	Up	4.2562	0.0026
hsa_circ_RSBN1L_11059	7	exon	RSBN1L	Up	4.2336	0.0028
hsa_circ_0051427	19	exon	RELB	Up	4.1916	0.0032
hsa_circ_0004188	18	intron	PTPRM	Up	3.6088	0.0135
hsa_circ_ACTN1_2453	14	exon	ACTN1	Up	3.602	0.0137
hsa_circ_0063534	22	exon	RANGAP1	Up	1.6825	0.0035
hsa_circ_0001860	9	exon	ZCCHC7	Down	−6.7445	0.0000
hsa_circ_0004539	5	exon	LHFPL2	Down	−5.6548	0.0000
hsa_circ_0008585	2	exon	BIRC6	Down	−5.3167	0.0000
hsa_circ_0004161	1	exon	DPYD	Down	−4.8136	0.0003
hsa_circ_0002127	11	exon	HIPK3	Down	−4.7746	0.0004
hsa_circ_MAD1L1_10741	7	exon	AC069288.1; MAD1L1	Down	−4.7053	0.0005
hsa_circ_0005395	2	exon	NBEAL1	Down	−4.2523	0.0024
hsa_circ_0001491	5	exon	IPO11	Down	−4.2405	0.0025
hsa_circ_XPO7_11370	8	exon	XPO7	Down	−4.2344	0.0025
hsa_circ_0005564	8	exon	FGFR1	Down	−2.413	0.0250

### 3.2 Enrichment analysis of the GO and KEGG pathways for differentially expressed circRNAs

**FIGURE 2:**
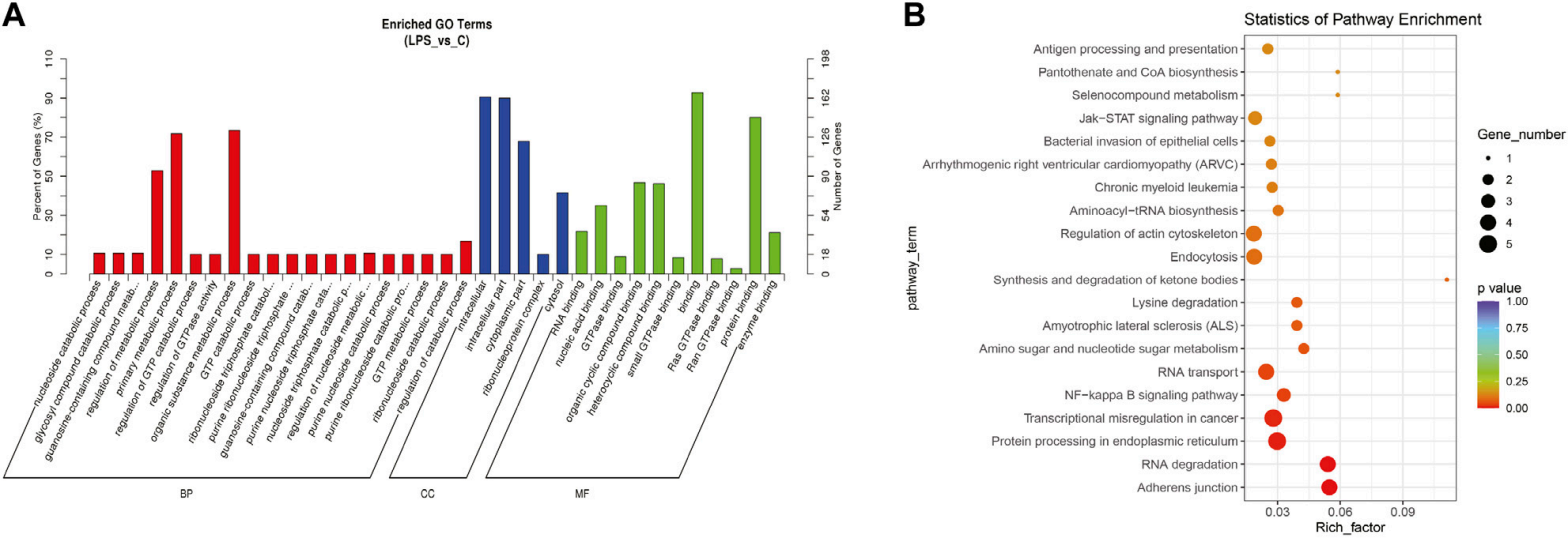
GO and KEGG pathway enrichment analyses for DE circRNAs. **(A)** The top significantly enriched GO terms for DE circRNAs. The horizontal axis represents the name of the GO term, and the vertical axis represents the number of genes. **(B)** The top 20 significantly enriched KEGG pathways. The size of the dots represents the number of DE circRNAs enriched in the pathway, and the color of the dots represents the *p*-value. The horizontal axis shows the rich factor (the degree of enrichment of DE circRNAs), and the vertical axis shows the pathway names.

The top significantly enriched GO terms of DE circRNAs were shown in Figure 2A, the results showed that the primary metabolic process and the organic substance metabolic process were more enriched for BP, and the intracellular and intracellular part had more enriched for CC, while binding was more enriched for MF. According to the analysis of KEGG pathway enrichment (Figure 2B), DE circRNAs were mainly enriched at the adherens junction, suggesting the significant associations of DE circRNAs with the endothelial barrier in LPS-induced sepsis, RNA degradation, and protein processing in the endoplasmic reticulum, *etc.* The adherens junction pathway was identified for our further study (Supplementary Figure 1S). The circRNAs associated with the adherens junction pathway were listed in Table 2, of which hsa_circ_0074158, hsa_circ_0004188, hsa_circ_0005564, and hsa_circ_ACTN1_2453 belonged to 203 DE circRNAs.

**TABLE 2:** CircRNAs related to the adherens junction pathway.

CircRNA_ID	Chromosome	Feature	Gene symbol	Regulation	log2FoldChange	*p*-value	DE circRNA
hsa_circ_0074158	5	exon	CTNNA1	Up	4.4764	0.0012	Yes
hsa_circ_0004188	18	intron	PTPRM	Up	3.6088	0.0135	Yes
hsa_circ_ACTN1_2453	14	exon	ACTN1	Up	3.602	0.0137	Yes
hsa_circ_0002872	18	exon	PTPRM	Up	1.8256	0.2126	No
hsa_circ_0074171	5	exon	CTNNA1	Up	1.5502	0.2802	No
hsa_circ_0046813	18	exon	PTPRM	Up	1.0413	0.4253	No
hsa_circ_0002913	14	exon	ACTN1	Up	0.85031	0.4615	No
hsa_circ_0032321	14	exon	ACTN1	Up	0.43822	0.7609	No
hsa_circ_0008196	5	exon	CTNNA1	Up	0.28868	0.8438	No
hsa_circ_0007440	5	exon	CTNNA1	Up	0.031718	0.9676	No
hsa_circ_0006114	18	exon	PTPRM	Down	−0.22938	0.7611	No
hsa_circ_0008016	8	exon	FGFR1	Down	−0.97928	0.1099	No
hsa_circ_PTPRM_4378	18	exon	PTPRM	Down	−1.4552	0.3111	No
hsa_circ_ACTN1_2450	14	exon	ACTN1	Down	−1.4768	0.3018	No
hsa_circ_0007644	14	exon	ACTN1	Down	−1.7358	0.2436	No
hsa_circ_0005564	8	exon	FGFR1	Down	−2.413	0.0250	Yes

### 3.3 Analysis of the ceRNA regulatory networks

**FIGURE 3:**
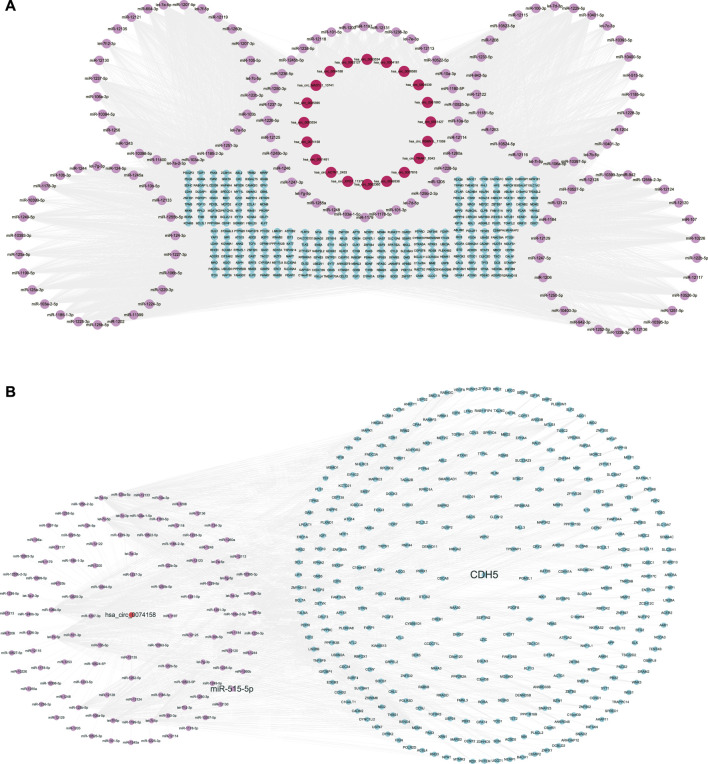
CeRNA regulatory networks. **(A)** CircRNA-miRNA-mRNA network of the 20 DE circRNAs. **(B)** CircRNA-miRNA-mRNA network of hsa_circ_0074158. Red represents circRNA, pink represents miRNA, and blue represents mRNA.

We constructed the ceRNA regulatory networks analysis to elucidate the relationship in sepsis using circRNA-miRNA-mRNA. 138 target miRNAs for 20 DE circRNAs and 21307 target mRNAs for the miRNAs were predicted. We selected top target mRNAs of the 20 DE circRNAs to conduct a circRNA-miRNA-mRNA network (Figure 3A). The network showed that CDH5 (VE-cadherin) is the target of hsa-miR-515-5p and hsa-miR-515-5p is the target of hsa_circ_0074158. To select hsa_circ_0074158 for further study, we also conducted a circRNA-miRNA-mRNA network for hsa_circ_0074158 (Figure 3B).

### 3.4 Validation of the RNA-seq results and demographic information of hsa_circ_0074158

**FIGURE 4:**
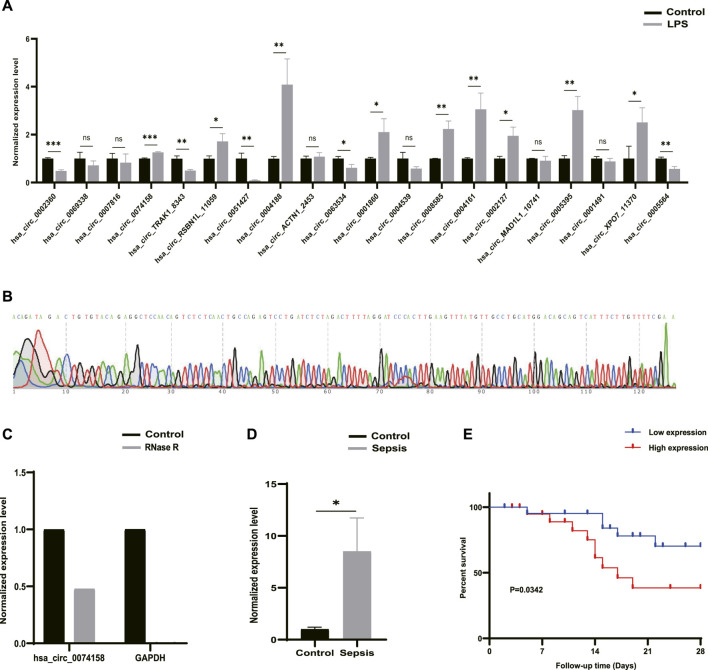
Candidate circRNAs. **(A)** Validation of 20 DE circRNAs expression by RT-qPCR. **(B)** Sanger sequencing confirmed the back splice junction site of hsa_circ_0074158. Green stands for A, red stands for T, black stands for G, and blue stands for C .**(C)** RNase R treatment experiments. **(D)** Validation of the hsa_circ_0074158 expression in the whole blood of 44 patients with sepsis and 48 healthy individuals. **(E)** Kaplan-Meier survival curve of patients with sepsis. *p < 0.05, **p < 0.01, ***p < 0.001. Control: Control groups. LPS: LPS groups. RNase R: RNase R treatment groups. Sepsis: Sepsis groups.

RT-qPCR was performed to verify the 20 DE circRNAs identified by RNA-seq analysis above. Combined with log_2_FoldChange in Table 1, the results showed that only the expression patterns of hsa_circ_0074158, hsa_circ_RSBN1L_11059, hsa_circ_0004188, and hsa_circ_0005564 were highly consistent with the RNA-seq results (Figure 4A). The hsa_circ_0074158 was selected for our further study according to the expression level and KEGG pathway enrichment analysis. To verify the splice site of hsa_circ_0074158, the RT-qPCR amplification and Sanger sequencing were performed and the results confirmed the back splice junction site (Figure 4B). The linear RNAs were removed using an RNase R treatment experiment (Epicentre Technologies, United States) (Figure 4C). The RT-qPCR results of 92 patients showed that the expression pattern of hsa_circ_0074158 was highly consistent with the results of the RNA-seq (Figure 4D). We also collected demographic information on sepsis patients. Based on the median expression of hsa_circ_0074158, we classified the 44 patients into a low expression group (*n* = 22) and a high expression group (*n* = 22). We found that the expression level of hsa_circ_0074158 was significantly correlated with chronic comorbidities (Table 3). Additionally, survival analyses were also performed based on the expression levels of hsa_circ_0074158. The Kaplan-Meier method with the log-rank test was used to assess the survival rate of sepsis patients (Figure 4E). And the analysis showed that the patients of the hsa_circ_0074158 high expression group exhibited poor overall survival (*p =* 0.0342) compared to those of the hsa_circ_0074158 low expression group.

**TABLE 3:** Demographic information for sepsis patients according to hsa_circ_0074158 expression.

Parameters	Low expression	High expression	*p*-Value
Gender			0.540
Male	12	14	
Female	10	8	
Age (years)			0.093
≥ 60	17	21	
< 60	5	1	
Infection souce			0.793
Lung	13	10	
Urinary tract	5	5	
Skin or soft tissues	1	1	
Other	3	6	
Comorbidities			0.015
Yes	13	20	
No	9	2	

### 3.5 Role of hsa_circ_0074158 in sepsis-induced endothelial barrier disruption

**FIGURE 5:**
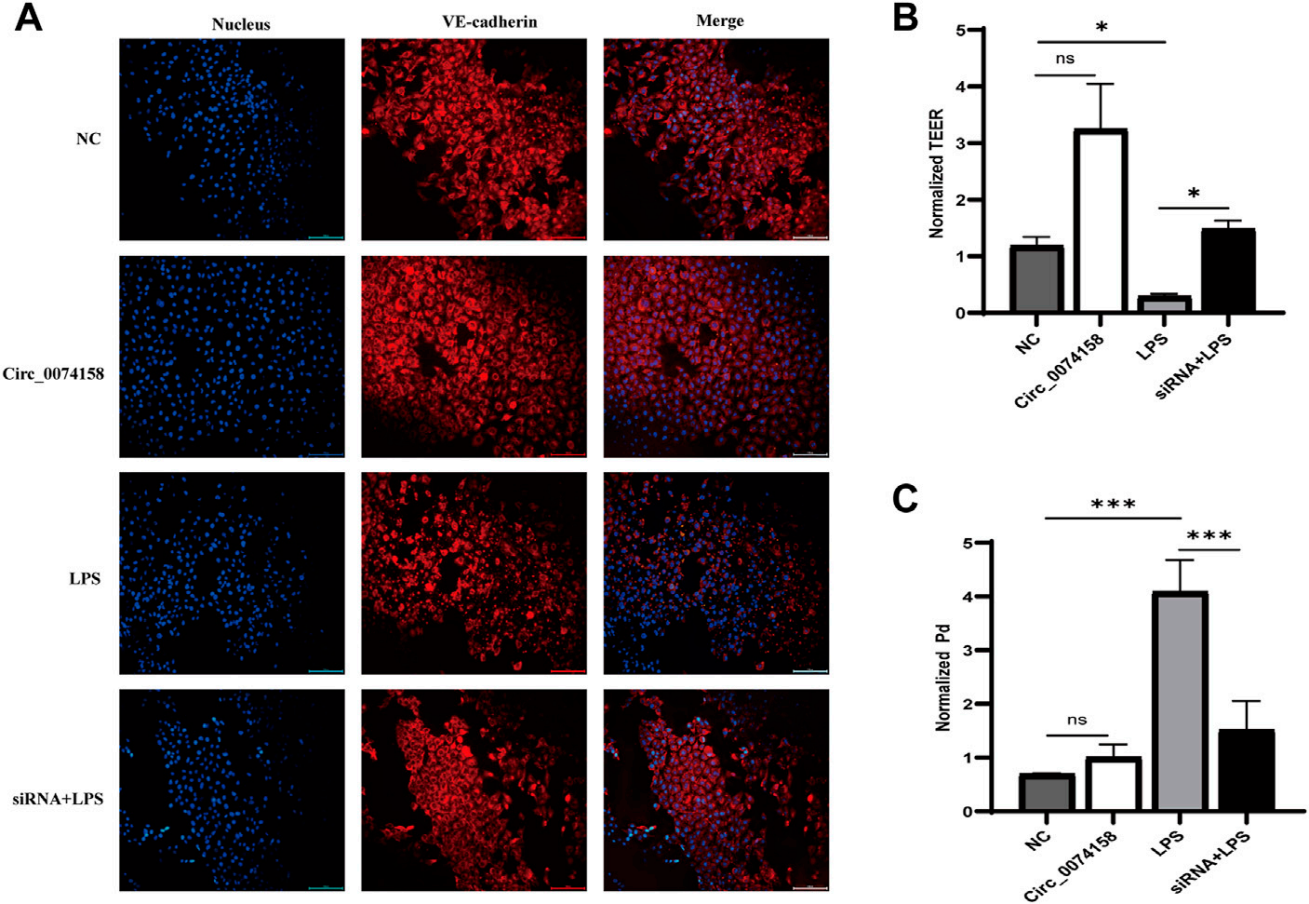
Hsa_circ_0074158 regulates the endothelial barrier function in sepsis. **(A)** The silencing of hsa_circ_0074158 enhanced the expression of VE-cadherin, which was down-regulated in LPS-induced sepsis. Bar = 100 µm. **(B)** The endothelial permeability was measured by TEER assay and showed. **(C)** The endothelial permeability was determined using the FITC-dextran assay and showed. *p < 0.05, **p < 0.01, ***p < 0.001. NC: Negative control groups. LPS: LPS groups. Circ_0074158: Circ_0074158 groups. siRNA + LPS: si-circ_0074158 + LPS groups.

To illustrate the effects of hsa_circ_0074158 on the endothelial barrier function in sepsis, siRNA was used to confirm the role. Previous research has shown that LPS can cause VE-cadherin disruption and lead to adherens junction disruption (Chan et al., 2019). In this study, immunofluorescence staining demonstrated that hsa_circ_0074158 knockdown can significantly enhance the expression of VE-cadherin, which was down-regulated in LPS-induced sepsis (Figure 5A). Experiments also showed that knockdown of hsa_circ_0074158 could increase TEER (Figure 5B) and decrease the extravasation of FITC-dextran (Figure 5C). In summary, these results fully confirmed that si-circ_0074158 reversed LPS-induced suppression of VE-cadherin and endothelial hyperpermeability, mimicking barrier protection.

## 4 Discussion

With the development of bioinformatic techniques and approaches, the associations of circRNA with diseases, such as cancer, cardiovascular disease, nervous system disease, immune-related disorders, and infections, were identified and verified (Chen et al., 2018; Wang et al., 2018; He et al., 2019; Hosaka et al., 2019; Wang et al., 2019). The differential expression of circRNA plays an important role in the diagnosis, treatment, and prognosis of the disease. Sepsis is characterized by complex pathogenesis, atypical clinical manifestations, and a difficult diagnosis. Despite the significant advances that have been made in treatment technologies, sepsis remains a serious clinical syndrome with substantial morbidity and mortality. To further clarify the pathogenesis and provide a novel approach for the diagnosis and treatment of sepsis, we explored the transcriptome characteristics of LPS-induced sepsis by RNA-seq.

Herein, 203 significantly DE circRNAs, including 77 up-regulated and 126 down-regulated, were identified. Then 20 DE circRNAs were selected for RT-qPCR validation. According to the results from the RNA-seq analysis and filtering conditions, only the expression of hsa_circ_0074158, hsa_circ_RSBN1L_11059, hsa_circ_0004188, and hsa_circ_0005564 were highly consistent with the results of the RNA-seq, which indicated that these circRNAs were potential biomarkers and drug targets for sepsis treatment. However, more research is needed to validate the results.

We performed GO and KEGG pathway enrichment analyses to explore the potential pathophysiological mechanism in sepsis. The results of the GO enrichment analysis showed that the most significantly enriched GO term in the BF was organic substance metabolic process, the CC was intracellular, and in MF, binding. The GO enrichment analysis indicated that circRNA functions were highly associated with the metabolic process and GTPase, which have been shown to have a relationship with sepsis and endothelial barrier function in previous studies, respectively (Mira et al., 2017; Aslam et al., 2019; Sanchez and Raja, 2022). The enrichment analysis of the KEGG pathway revealed that circRNAs were mainly involved in adherens junction, NF-kappa B signaling pathway, endocytosis, regulation of actin cytoskeleton, bacterial invasion of epithelial cells, antigen processing and presentation, and which also predicted that circRNAs might be related to endothelial barrier function in sepsis (Garcia et al., 2018; McRae et al., 2018; Radeva and Waschke, 2018; Leonard et al., 2019; Strauss and Gourdie, 2020).

We also constructed ceRNA regulatory networks to reveal the regulatory mechanism in sepsis. According to the ceRNA mechanism, there are 138 target miRNAs for 20 DE circRNAs and 21307 target mRNAs for the miRNAs were identified. In particular, CDH5 (VE-cadherin) is the target gene of hsa-miR-515-5p and hsa-miR-515-5p is the target of hsa_circ_0074158. The KEGG pathway analysis showed that hsa_circ_0074158 was related to the adherens junction. As we know, adherens junctions are specialized forms of adhesive contacts based on VE-cadherin, and adherens junctions are mainly composed of VE-cadherin (Dejana et al., 2008). Tight junctions, adherens junctions, and gaps are more closely correlated with the function of the endothelial barrier. The endothelial barrier function plays a key role in the development of sepsis. Therefore, we hypothesized that hsa_circ_0074158 regulated VE-cadherin and endothelial barrier function in sepsis through hsa-miR-515-5p. We also evaluated the adherens function of the endothelial barrier using VE-cadherin expression.

The hsa_circ_0074158 in the KEGG pathway of the adherens junction was selected for further study. High consistency of the *in vivo* RT-qPCR results with the RNA-seq analysis was observed. We used a combination of RNase R treatment and Sanger sequencing to confirm the cyclization feature of hsa_circ_0074158. Immunofluorescence assay and endothelial permeability demonstrated that LPS-induced sepsis can disrupt VE-cadherin expression and enhance endothelial permeability, while knockdown of hsa_circ_0074158 can suppress the sepsis-caused aberrant regulation. Hence, we hypothesized that hsa_circ_0074158 was potentially involved in endothelial barrier dysfunction. However, these results are preliminary and further confirmation with an expanded study is needed.

Recently, an increasing number of studies have investigated the function of circRNAs. It is well known that circRNAs can act as miRNA sponges, thus regulating the expression of miRNAs (Thomson and Dinger, 2016; Panda, 2018). Many studies also demonstrated that circRNAs might be used as potential biomarkers and therapeutic targets for the treatment and therapy of sepsis. A clinical study demonstrated that circ-PRKCI was an independent factor to predict the mortality risk of sepsis patients (Wei and Yu, 2020). Another clinical study that included 25 patients with sepsis observed the differential expression of hsa_circRNA_104484 and hsa_circRNA_104670 in serum exosomes, indicating the potential of hsa_circRNA_104484 and hsa_circRNA_104670 as a biomarker and potential therapeutic target (Wei and Yu, 2020). A recent review analyzed the biological characteristics of circRNAs in sepsis based on the Medline database (Wei et al., 2022). In our study, survival analyses showed that people with high hsa_circ_0074158 expression have higher mortality, suggesting that hsa_circ_0074158 could be used as a biomarker. To date, more and more attention has been paid to the biomarkers of sepsis, which can help to diagnose, evaluate the disease, and judge the prognosis. However, the ideal biomarker for sepsis has not yet been determined. To prevent, identify, diagnose, and treat sepsis early, the combination of known biomarkers for sepsis may be more effective. If there are biomarkers that can be used to predict sepsis early, mortality and the use of unnecessary antibiotics can be reduced. The identification of the biomarkers for the prediction of sepsis has become one of the hotspots of research. In the future, when searching for sepsis biomarkers, we should pay more attention to early prevention of sepsis, which can reduce the morbidity and mortality of sepsis more efficiently. Furthermore, our research also showed that circRNA was potentially associated with endothelial barrier function in sepsis, which may provide more possibilities for the prevention of sepsis in advance.

## 5 Conclusion

In our present study, we clarified the differential expression and potential functions of circRNAs in LPS-induced sepsis. CirculaRNAs, including hsa_circ_0074158, hsa_circ_RSBN1L_11059, hsa_circ_0004188, and hsa_circ_0005564, could serve as potential biomarkers and therapeutic targets for sepsis treatment and therapy. Meanwhile, we observed significant associations of hsa_circ_0074158 with the endothelial barrier function in sepsis. The results also showed that the knockdown of hsa_circ_0074158 could significantly improved VE-cadherin expression, reduced endothelial permeability, and subsequently protect the adherens junction in sepsis. Based on ceRNA regulatory network, we hypothesized that hsa_circ_0074158 regulated the endothelial barrier disruption in sepsis through hsa-miR-515-5p. Collectively, our data demonstrated that dysregulation of circRNAs was significantly associated with sepsis and circRNAs played a potential role in sepsis, suggesting the role of circRNA as a promising biomarker and the significant clinical values for sepsis treatment and therapy.

## Data Availability

The datasets presented in this study can be found in online repositories. The names of the repository/repositories and accession number(s) can be found below: https://www.ncbi.nlm.nih.gov/geo/, GSE204776.

## Abbreviations

CircRNAs, Circular RNAs; RT-qPCR, Quantitative real-time polymerase chain reaction; GO, Gene Ontology; KEGG, Kyoto Encyclopedia of Genes and Genomes; CeRNA, Competitive endogenous RNA; DE, Differentially expressed; VE-cadherin, Vascular endothelial cadherin; TEER, Transendothelial electrical resistance; FITC, Fluorescein isothiocyanate; LPS, Lipopolysaccharide; HUVECs, Human umbilical vein endothelial cells; DMEM, Dulbecco’s modified Eagle’s Medium; FBS, Fetal bovine serum; PBS, Phosphate buffer saline; RNA-seq, RNA-sequencing; RNase, Ribonuclease; BP, Biological process; CC, Cellular component; MF, Molecular function.

## References

[B1] Abdelmohsen K. Panda A. C. Munk R. Grammatikakis I. Dudekula D. B. De S. (2017). Identification of HuR target circular RNAs uncovers suppression of PABPN1 translation by CircPABPN1. RNA Biol. 14 (3), 361–369. 10.1080/15476286.2017.1279788 28080204 PMC5367248

[B2] Agarwal V. Bell G. W. Nam J. W. (2015). Predicting effective microRNA target sites in mammalian mRNAs[J]. Cambridgeshire, United Kingdom: eLife Sciences Publications Ltd. 4. 10.7554/eLife.05005 PMC4532895 26267216

[B3] Ashwal-Fluss R. Meyer M. Pamudurti N. R. Ivanov A. Bartok O. Hanan M. (2014). circRNA biogenesis competes with pre-mRNA splicing. Mol. Cell 56 (1), 55–66. 10.1016/j.molcel.2014.08.019 25242144

[B4] Aslam M. Troidl C. Tanislav C. Rohrbach S. Gunduz D. Hamm C. W. (2019). Inhibition of protein prenylation of GTPases alters endothelial barrier function. Int. J. Mol. Sci 21 (1), E2. 10.3390/ijms21010002 31861297 PMC6981884

[B5] Beltran-Garcia J. Osca-Verdegal R. Nacher-Sendra E. Pallardo F. V. Garcia-Gimenez J. L. (2020). Circular RNAs in sepsis: Biogenesis, function, and clinical significance. Cells 9 (6), E1544. 10.3390/cells9061544 32630422 PMC7349763

[B6] Brasch J. Harrison O. J. Ahlsen G. Carnally S. M. Henderson R. M. Honig B. (2011). Structure and binding mechanism of vascular endothelial cadherin: A divergent classical cadherin. J. Mol. Biol. 408 (1), 57–73. 10.1016/j.jmb.2011.01.031 21269602 PMC3084036

[B7] Chan Y. H. Harith H. H. Israf D. A. Tham C. L. (2019). Differential regulation of LPS-mediated VE-cadherin disruption in human endothelial cells and the underlying signaling pathways: A mini review. Front. Cell Dev. Biol. 7, 280. 10.3389/fcell.2019.00280 31970155 PMC6955238

[B8] Chen B. Wei W. Huang X. Xie X. Kong Y. Dai D. (2018). circEPSTI1 as a prognostic marker and mediator of triple-negative breast cancer progression. Theranostics 8 (14), 4003–4015. 10.7150/thno.24106 30083277 PMC6071524

[B9] Chen L. Huang C. Shan G. (2021). Circular RNAs in physiology and non-immunological diseases. Trends biochem. Sci. 47, 250–264. 10.1016/j.tibs.2021.11.004 34865956

[B10] Chen L. L. (2016). The biogenesis and emerging roles of circular RNAs. Nat. Rev. Mol. Cell Biol. 17 (4), 205–211. 10.1038/nrm.2015.32 26908011

[B11] Chen L. (2020). The expanding regulatory mechanisms and cellular functions of circular RNAs. Nat. Rev. Mol. Cell Biol. 21 (8), 475–490. 10.1038/s41580-020-0243-y 32366901

[B12] Cocquerelle C. Daubersies P. Majerus M. A. Kerckaert J. P. Bailleul B. (1992). Splicing with inverted order of exons occurs proximal to large introns. EMBO J. 11 (3), 1095–1098. 10.1002/j.1460-2075.1992.tb05148.x 1339341 PMC556550

[B13] Cocquerelle C. Mascrez B. Hetuin D. Bailleul B. (1993). Mis-splicing yields circular RNA molecules. FASEB J. 7 (1), 155–160. 10.1096/fasebj.7.1.7678559 7678559

[B14] Consortium T. G. O. (2019). The gene Ontology resource: 20 years and still GOing strong. Nucleic Acids Res. 47 (D1), D330–D338. 10.1093/nar/gky1055 30395331 PMC6323945

[B15] Dejana E. Orsenigo F. Lampugnani M. G. (2008). The role of adherens junctions and VE-cadherin in the control of vascular permeability. J. Cell Sci. 121 (13), 2115–2122. 10.1242/jcs.017897 18565824

[B16] Fleischmann C. Scherag A. Adhikari N. K. Hartog C. S. Tsaganos T. Schlattmann P. (2016). Assessment of global incidence and mortality of hospital-treated sepsis. Current estimates and limitations. Am. J. Respir. Crit. Care Med. 193 (3), 259–272. 10.1164/rccm.201504-0781OC 26414292

[B17] Garcia M. A. Nelson W. J. Chavez N. (2018). Cell-cell junctions organize structural and signaling networks. Cold Spring Harb. Perspect. Biol. 10 (4), a029181. 10.1101/cshperspect.a029181 28600395 PMC5773398

[B18] Godo S. Shimokawa H. (2017). Endothelial functions. Arterioscler. Thromb. Vasc. Biol. 37 (9), e108–e114. 10.1161/ATVBAHA.117.309813 28835487

[B19] Godo S. Takahashi J. Yasuda S. Shimokawa H. (2021). Endothelium in coronary macrovascular and microvascular diseases. J. Cardiovasc. Pharmacol. 78 (6), S19–S29. 10.1097/FJC.0000000000001089 34840261 PMC8647695

[B20] He J. Ren M. Li H. Yang L. Wang X. Yang Q. (2019). Exosomal circular RNA as a biomarker platform for the early diagnosis of immune-mediated demyelinating disease. Front. Genet. 10, 860. 10.3389/fgene.2019.00860 31611906 PMC6777646

[B21] Hosaka T. Yamashita T. Tamaoka A. Kwak S. (2019). Extracellular RNAs as biomarkers of sporadic amyotrophic lateral sclerosis and other neurodegenerative diseases. Int. J. Mol. Sci. 20 (13), E3148. 10.3390/ijms20133148 31252669 PMC6651127

[B22] Hsu M. T. Coca-Prados M. (1979). Electron microscopic evidence for the circular form of RNA in the cytoplasm of eukaryotic cells. Nature 280 (5720), 339–340. 10.1038/280339a0 460409

[B23] Huang Y. Zhang C. Xiong J. Ren H. (2021). Emerging important roles of circRNAs in human cancer and other diseases. Genes Dis. 8 (4), 412–423. 10.1016/j.gendis.2020.07.012 34179306 PMC8209354

[B24] Jiang B. Zhang J. Sun X. Yang C. Cheng G. Xu M. (2022). Circulating exosomal hsa_circRNA_0039480 is highly expressed in gestational diabetes mellitus and may be served as a biomarker for early diagnosis of GDM. J. Transl. Med. 20 (1), 5. 10.1186/s12967-021-03195-5 34980149 PMC8722188

[B25] Kanehisa M. Goto S. (2000). Kegg: Kyoto encyclopedia of genes and genomes. Nucleic Acids Res. 28 (1), 27–30. 10.1093/nar/28.1.27 10592173 PMC102409

[B26] Kim D. Langmead B. Salzberg S. L. (2015). Hisat: A fast spliced aligner with low memory requirements. Nat. Methods 12 (4), 357–360. 10.1038/nmeth.3317 25751142 PMC4655817

[B27] Kristensen L. S. Andersen M. S. Stagsted L. Ebbesen K. K. Hansen T. B. Kjems J. (2019). The biogenesis, biology and characterization of circular RNAs. Nat. Rev. Genet. 20 (11), 675–691. 10.1038/s41576-019-0158-7 31395983

[B28] Lee W. L. Slutsky A. S. (2010). Sepsis and endothelial permeability. N. Engl. J. Med. 363 (7), 689–691. 10.1056/NEJMcibr1007320 20818861

[B29] Leonard A. Millar M. W. Slavin S. A. Bijli K. M. Dionisio Santos D. A. Dean D. A. (2019). Critical role of autophagy regulator Beclin1 in endothelial cell inflammation and barrier disruption. Cell. Signal. 61, 120–129. 10.1016/j.cellsig.2019.04.013 31054328 PMC6685427

[B30] Li J. H. Liu S. Zhou H. Qu L. H. Yang J. H. (2014). starBase v2.0: decoding miRNA-ceRNA, miRNA-ncRNA and protein-RNA interaction networks from large-scale CLIP-Seq data. Nucleic Acids Res. 42 D92–D97. 10.1093/nar/gkt1248 24297251 PMC3964941

[B31] Love M. I. Huber W. Anders S. (2014). Moderated estimation of fold change and dispersion for RNA-seq data with DESeq2. Genome Biol. 15 (12), 550. 10.1186/s13059-014-0550-8 25516281 PMC4302049

[B32] Machado F. R. Cavalcanti A. B. Bozza F. A. Ferreira E. M. Angotti Carrara F. S. Sousa J. L. (2017). The epidemiology of sepsis in Brazilian intensive care units (the sepsis PREvalence assessment database, SPREAD): An observational study. Lancet. Infect. Dis. 17 (11), 1180–1189. 10.1016/S1473-3099(17)30322-5 28826588

[B33] Martin-Fernandez M. Tamayo-Velasco A. Aller R. Gonzalo-Benito H. Martinez-Paz P. (2021). Endothelial dysfunction and neutrophil degranulation as central events in sepsis physiopathology. Int. J. Mol. Sci. 22 (12), 6272. 10.3390/ijms22126272 34200950 PMC8230689

[B34] McRae M. LaFratta L. M. Nguyen B. M. Paris J. J. Hauser K. F. Conway D. E. (2018). Characterization of cell-cell junction changes associated with the formation of a strong endothelial barrier. Tissue Barriers 6 (1), e1405774. 10.1080/21688370.2017.1405774 29388870 PMC5823545

[B35] Mira J. C. Gentile L. F. Mathias B. J. Efron P. A. Brakenridge S. C. Mohr A. M. (2017). Sepsis pathophysiology, chronic critical illness, and persistent inflammation-immunosuppression and catabolism syndrome. Crit. Care Med. 45 (2), 253–262. 10.1097/CCM.0000000000002074 27632674 PMC5243156

[B36] Nava E. Llorens S. (2019). The local regulation of vascular function: From an inside-outside to an outside-inside model. Front. Physiol. 10, 729. 10.3389/fphys.2019.00729 31244683 PMC6581701

[B37] Nigro J. M. Cho K. R. Fearon E. R. Kern S. E. Ruppert J. M. Oliner J. D. (1991). Scrambled exons. Cell 64 (3), 607–613. 10.1016/0092-8674(91)90244-s 1991322

[B38] Panda A. C. (2018). Circular RNAs act as miRNA sponges. Adv. Exp. Med. Biol. 1087, 67–79. 10.1007/978-981-13-1426-1_6 30259358

[B39] Pasman Z. Been M. D. Garcia-Blanco M. A. (1996). Exon circularization in mammalian nuclear extracts. RNA 2 (6), 603–610. 8718689 PMC1369399

[B40] Radeva M. Y. Waschke J. (2018). Mind the gap: Mechanisms regulating the endothelial barrier. Acta Physiol. 222 (1), e12860. 10.1111/apha.12860 28231640

[B41] Rajendran P. Rengarajan T. Thangavel J. Nishigaki Y. Sakthisekaran D. Sethi G. (2013). The vascular endothelium and human diseases. Int. J. Biol. Sci. 9 (10), 1057–1069. 10.7150/ijbs.7502 24250251 PMC3831119

[B42] Sanchez L. D. N. A. Raja A. (2022). , Metabolism physiology,National Library of Medicine Bethesda, MD, USA

[B43] Sanger H. L. Klotz G. Riesner D. Gross H. J. Kleinschmidt A. K. (1976). Viroids are single-stranded covalently closed circular RNA molecules existing as highly base-paired rod-like structures. Proc. Natl. Acad. Sci. U. S. A. 73 (11), 3852–3856. 10.1073/pnas.73.11.3852 1069269 PMC431239

[B44] Shimokawa H. Godo S. (2020). Nitric oxide and endothelium-dependent hyperpolarization mediated by hydrogen peroxide in health and disease. Basic Clin. Pharmacol. Toxicol. 127 (2), 92–101. 10.1111/bcpt.13377 31846200

[B45] Singer M. Deutschman C. S. Seymour C. W. Shankar-Hari M. Annane D. Bauer M. (2016). The third international consensus definitions for sepsis and septic shock (Sepsis-3). JAMA 315 (8), 801–810. 10.1001/jama.2016.0287 26903338 PMC4968574

[B46] Sticht C. De La Torre C. Parveen A. Gretz N. (2018). miRWalk: An online resource for prediction of microRNA binding sites. PLoS One 13 (10), e0206239. 10.1371/journal.pone.0206239 30335862 PMC6193719

[B47] Strauss R. E. Gourdie R. G. (2020). Cx43 and the actin cytoskeleton: Novel roles and implications for cell-cell junction-based barrier function regulation. Biomolecules 10 (12), E1656. 10.3390/biom10121656 33321985 PMC7764618

[B48] Thomson D. W. Dinger M. E. (2016). Endogenous microRNA sponges: Evidence and controversy. Nat. Rev. Genet. 17 (5), 272–283. 10.1038/nrg.2016.20 27040487

[B49] Tian C. Liu J. Di X. Cong S. Zhao M. Wang K. (2021). Exosomal hsa_circRNA_104484 and hsa_circRNA_104670 may serve as potential novel biomarkers and therapeutic targets for sepsis. Sci. Rep. 11 (1), 14141. 10.1038/s41598-021-93246-0 34238972 PMC8266806

[B50] Verduci L. Tarcitano E. Strano S. Yarden Y. Blandino G. (2021). CircRNAs: Role in human diseases and potential use as biomarkers. Cell Death Dis. 12 (5), 468. 10.1038/s41419-021-03743-3 33976116 PMC8113373

[B51] Vincent J. L. Marshall J. C. Namendys-Silva S. A. Francois B. Martin-Loeches I. Lipman J. (2014). Assessment of the worldwide burden of critical illness: The intensive care over nations (ICON) audit. Lancet. Respir. Med. 2 (5), 380–386. 10.1016/S2213-2600(14)70061-X 24740011

[B52] Wang Q. Wang J. Xin Y. He Z. Zhou X. Liu X. (2022). Hsa_circ_0005729 enhances the accuracy in diagnosing parathyroid carcinoma[J]. Endocr Connect,Bethesda, MD, USA 11(2) 10.1530/EC-21-0605 PMC8859958 35029546

[B53] Wang R. Zhang S. Chen X. Li N. Li J. Jia R. (2018). CircNT5E acts as a sponge of miR-422a to promote glioblastoma tumorigenesis. Cancer Res. 78 (17), 4812–4825. 10.1158/0008-5472.CAN-18-0532 29967262

[B54] Wang W. Wang Y. Piao H. Li B. Huang M. Zhu Z. (2019). Circular RNAs as potential biomarkers and therapeutics for cardiovascular disease. PeerJ 7, e6831. 10.7717/peerj.6831 31119072 PMC6511224

[B55] Wei B. Yu L. (2020). Circular RNA PRKCI and microRNA-545 relate to sepsis risk, disease severity and 28-day mortality. Scand. J. Clin. Lab. Invest. 80 (8), 659–666. 10.1080/00365513.2020.1827291 32985287

[B56] Wei L. Yang Y. Wang W. Xu R. (2022). Circular RNAs in the pathogenesis of sepsis and their clinical implications: A narrative review. Ann. Acad. Med. Singap. 51 (4), 221–227. 10.47102/annals-acadmedsg.2021405 35506405

[B57] Wu J. Deng Z. Sun M. Zhang W. Yang Y. Zeng Z. (2020). Polydatin protects against lipopolysaccharide-induced endothelial barrier disruption via SIRT3 activation. Lab. Invest. 100 (4), 643–656. 10.1038/s41374-019-0332-8 31641228

[B58] Zhang P. Sun H. Wen P. Wang Y. Cui Y. Wu J. (2022). circRNA circMED27 acts as a prognostic factor and mediator to promote lenvatinib resistance of hepatocellular carcinoma. Mol. Ther. Nucleic Acids 27, 293–303. 10.1016/j.omtn.2021.12.001 35024242 PMC8718824

[B59] Zhong S. Wang J. Zhang Q. Xu H. Feng J. (2018). CircPrimer: A software for annotating circRNAs and determining the specificity of circRNA primers. BMC Bioinforma. 19 (1), 292. 10.1186/s12859-018-2304-1 PMC6090782 30075703

