# Supplementary material for: Hsa_circ_0074158 regulates the endothelial barrier function in sepsis and its potential value as a biomarker
Source: Front Genet. 2022 Nov 8;13:1002344. doi: 10.3389/fgene.2022.1002344 (PMC9679418; doi:10.3389/fgene.2022.1002344)
Supplement: Supplementary file 1 [file Image1.pdf]

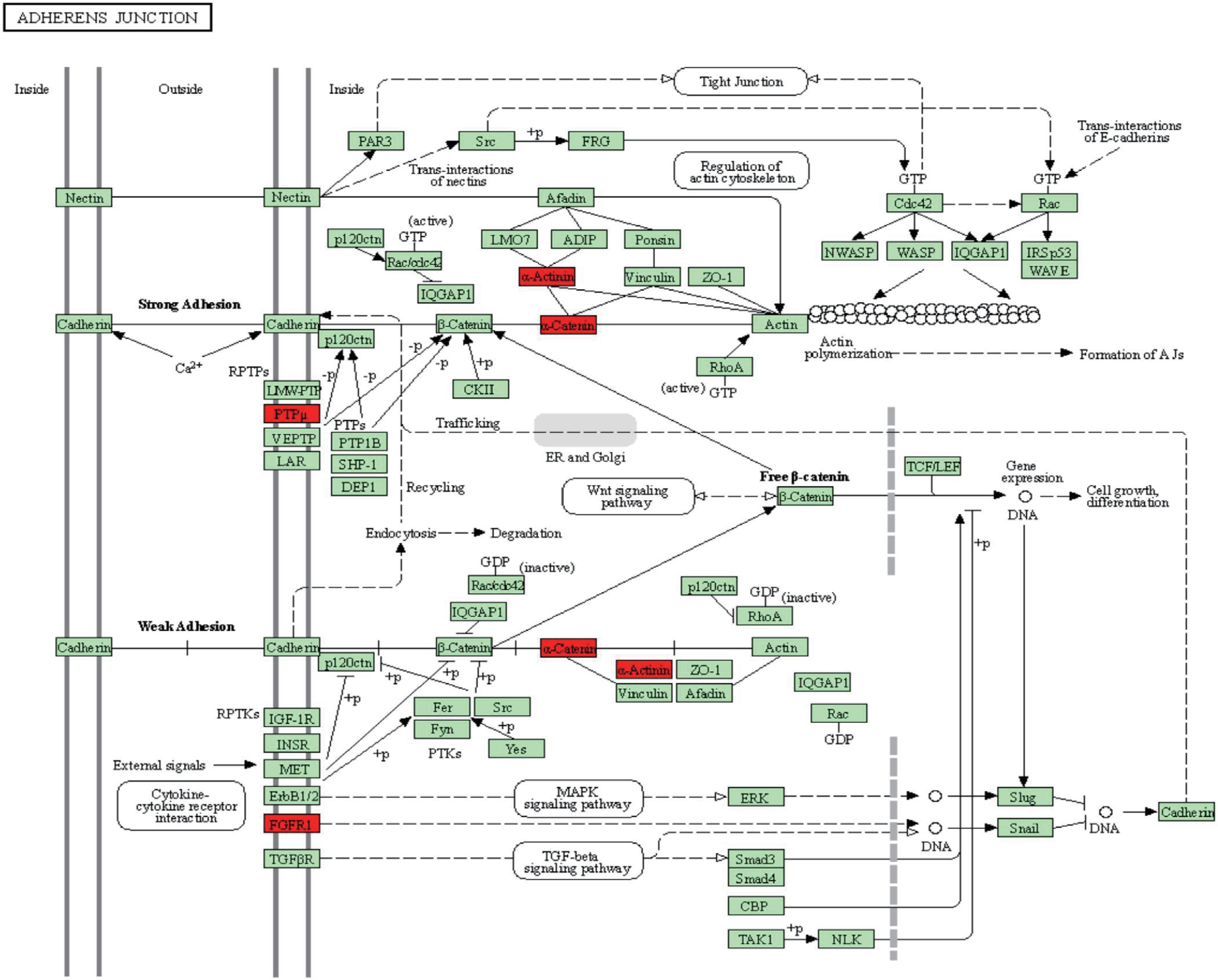

Supplementary Figure. Adherens junction pathway. The little boxes represent the host genes.
